# Regulatory effect of heat shock transcription factor-1 gene on heat shock proteins and its transcriptional regulation analysis in small abalone *Haliotis diversicolor*

**DOI:** 10.1186/s12860-020-00323-9

**Published:** 2020-11-24

**Authors:** Xin Zhang, Yuting Li, Yulong Sun, Mingxing Guo, Jianjun Feng, Yilei Wang, Ziping Zhang

**Affiliations:** 1 College of Animal Science, Fujian Agriculture and Forestry University, Fuzhou, 350002 China; 2 Fujian Engineering Research Center of Aquatic Breeding and Healthy Aquaculture, Xiamen, 361021 China; 3 Key Laboratory of Healthy Mariculture for the East China Sea, Ministry of Agriculture, Fisheries College, Jimei University, Xiamen, 361021 China; 4 Key Laboratory of Marine Biotechnology of Fujian Province, Institute of Oceanology, College of Animal Science, Fujian Agriculture and Forestry University, Fuzhou, 350002 China

**Keywords:** *Haliotis diversicolor*, Heat shock transcription factor-1, RNAi, Transcriptional regulation, GATA-1

## Abstract

**Background:**

The effects of diverse stresses ultimately alter the structures and functions of proteins. As molecular chaperones, heat shock proteins (HSPs) are a group of highly conserved proteins that help in the refolding of misfolded proteins and the elimination of irreversibly damaged proteins. They are mediated by a family of transcription factors called heat shock factors (HSFs). The small abalone *Haliotis diversicolor* is a species naturally distributed along the southern coast of China. In this study, the expression of *HdHSF1* was inhibited by RNAi in hemocytes in order to further elucidate the regulatory roles of *HdHSF1* on heat shock responsive genes in abalone. Meanwhile, to understand the transcriptional regulation of the *HdHSF1* gene, the 5′-upstream regulatory region of *HdHSF1* was characterized, and the relative promoter activity was examined by dual-luciferase reporter gene assay system in HEK293T cell lines.

**Results:**

After the inhibition of the *H. diversicolor HSF1* gene (*HdHSF1*) by dsRNA (double-stranded RNA), the expression of most heat shock related-genes was down-regulated (*p* < 0.05). It indicated the importance of *HdHSF1* in the heat shock response of *H. diversicolor*. Meanwhile, 5′-flanking region sequence (2633 bp) of the *HdHSF1* gene was cloned; it contained a putative core promoter region, TATA box, CAAT box, CpG island, and many transcription elements. In HEK293T cells, the 5′-flanking region sequence can drive expression of the enhanced green fluorescent protein (EGFP), proving its promoter function. Exposure of cells to the high-temperature (39 °C and 42 °C) resulted in the activation of *HdHSF1* promoter activity, which may explain why the expression of the *HdHSF1* gene participates in heat shock response. Luciferase activity of different recombinant plasmids, which contained different truncated promoter fragments of the *HdHSF1* gene in HEK293T cells, revealed the possible active regions of the promoter. To further identify the binding site of the critical transcription factor in the region, an expression vector with the site-directed mutation was constructed. After being mutated on the GATA-1 binding site, we found that the luciferase activity was significantly increased, which suggested that the GATA-1 binding site has a certain weakening effect on the activity of the *HdHSF1* promoter.

**Conclusions:**

These findings suggest that GATA-1 may be one of the transcription factors of *HdHSF1*, and a possible signaling pathway mediated by *HdHSF1* may exist in *H. diversicolor* to counteract the adverse effects of heat shock stress.

**Supplementary Information:**

**Supplementary information** accompanies this paper at 10.1186/s12860-020-00323-9.

## Background

Different stresses (for example, exposure to high temperature, hypoxia, heavy metals, and bacterial infections) can affect the structure and function of proteins [1]. The accumulation of denatured and aberrantly folded proteins enhances the synthesis of heat shock proteins (HSPs) that are a group of highly conserved proteins. They act as molecular chaperones by helping in the refolding of misfolded proteins and assisting in the elimination of irreversibly damaged proteins [2, 3]. Exposure to a multitude of stressors can activate the cell’s heat shock response (HSR). A family of transcription factors called heat shock factors (HSFs) bind to the heat shock elements (HSEs) that present in the promoter regions of HSP genes, mediates HSR and induces expression of HSPs [4]. Upon activation, each HSF undergoes extensive post-translational modifications and forms a transcriptionally active trimer that accumulates in the nucleus and acts on the target gene [5].

The HSF family consists of four different types: HSF1, HSF2, HSF3, and HSF4 [6]. HSF1, HSF2, and HSF4 had been identified in mammals, while HSF3 was described in chicken [7]. In vertebrates, HSF1 is thought to be the most important factor that induces thermal responses by regulating the refolding and assembly of HSPs, which are directly related to animal disease and life expectancy [8]. In invertebrates, HSF is required not only for the heat shock response but also for cell growth and differentiation and normal lifespan in yeast, *Caenorhabditis elegans*, and *Drosophila* [9–11]*.* HSF1 can drive the expression of a broad range of heat-responsive genes such as HSP90 in *Drosophila* during stress [12]. While several studies amply illustrate that HSP denaturation induces HSF1 expression, the exact molecular mechanisms about HSF1 transcriptional regulation remain unclear.

The small abalone *Haliotis diversicolor* is of great commercial value due to its unique nutrition and delicious taste [13]. However, the abalone industry has been severely affected by the frequent occurrence of infectious diseases and the deterioration of its environment, especially the hypoxia and thermal stress in hot summer months. These factors have threatened the abalone industry for a long time [14–17]. The high temperature in summer months along the southern coast can typically diminish the amount of dissolved oxygen, resulting in changes in metabolic and respiratory rates, and disease and high mortality of farmed abalones [16].

In the previous studies conducted by our team, several heat-shock related genes, such as heat-shock factor binding protein 1 (*HSBP1*), *HSP90*, and *HSF1* have been cloned and characterized from *H. diversicolor* [15]. Some other heat-shock related genes, such as *HSP22*, *HSP26*, *HSP60*, *HSP70*, *HSP105,* and *SIP*, have also been demonstrated to be up-regulated by thermal stress in hemocyte and hepatopancreas [18]. Moreover, the transcriptional regulation of *HdHSP90*, *HdHSC70*, and *HdHSP70* genes were all analyzed. The results indicated that HSEs were all presented in the 5′-flanking sequence of the three HSP genes, which can also be bound by HSF [19–21].

In this study, to further elucidate the regulatory effect of *HdHSF1* on other heat shock responsive genes in the small abalone, the expression of *HdHSF1* was inhibited by RNAi in hemocytes, and then the expression of this gene and other genes was assessed by quantitative real-time PCR (qRT-PCR). Meanwhile, to understand the transcriptional regulation of the *HdHSF1* gene, the 5′-upstream regulatory region of *HdHSF1* was characterized, and the relative promoter activity of sequential deletion constructs and site-directed mutagenesis construct containing the vital cis-acting element was examined by dual-luciferase reporter gene assay system in HEK293T cell lines. The findings will provide new insights into the regulation of *HSF1* expression and the mechanism of abalone to resist heat shock or other stresses.

## Results

### Expression of HSR related genes when the *HdHSF1* is inhibited by dsRNA

**Fig. 1:**
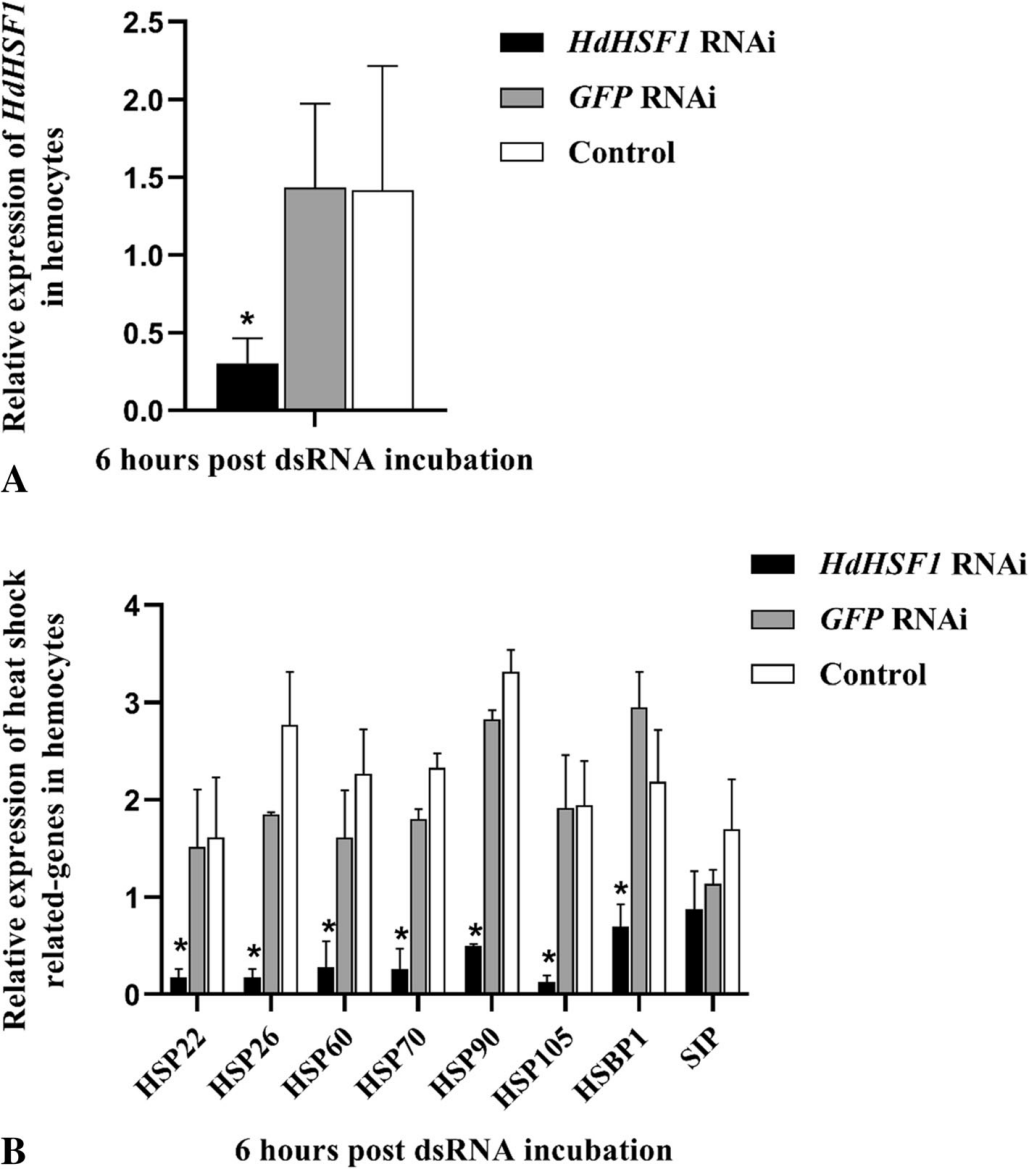
Expression analysis of the heat shock-related genes when the *HdHSF1* was inhibited by dsRNA in hemocytes. **a**. the mRNA expression level of the *HdHSF1* gene in the *Hd*HSF1 RNAi group was significantly downregulated compared with the *GFP* RNAi group and the blank control group (*p* < 0.05). **b**. the mRNA expression levels of 8 heat shock-related genes after the interference of *HdHSF1*. The X-axis represents treatment conditions and different target heat shock responsive transcripts. Y-axis represents the mRNA expression level of different genes. Six biological replicates were tested, and each sample was assayed in triplication. A significant difference between the experimental group and the control group was indicated by a (*) at *p* < 0.05. Control: blank control group. GFP-RNAi: group in which green fluorescent protein (GFP) gene was inhibited by dsRNA. HdHSF1-RNAi: group in which *HdHSF1* was inhibited by dsRNA

Several heat-shock related genes have been reported to be regulated by thermal stress in our previous studies [15, 18]. To further study the importance of the *HdHSF1* gene on these HSR genes, dsRNA (double-stranded RNA) was used to inhibit the expression of the *HdHSF1* gene. The expression of *HdHSF1* was tested by qRT-PCR, and the results showed that the gene expression in the experimental group was significantly decreased (*p* < 0.05) compared with the *GFP* RNAi group (control group) and the blank control group (Fig. 1 A). After the inhibition of *HdHSF1*, the expressions of *HSP22*, *HSP26*, *HSP60*, *HSP70*, *HSP90*, *HSP105,* and *HSBP1* were also significantly lower than those of the *GFP* RNAi group and blank control group (Fig. 1 B). However, the expression of *SIP* was not significantly affected by the interference of *HdHSF1* (*p* > 0.05).

### 5′ upstream sequences of *HdHSF1* gene

**Fig. 2:**
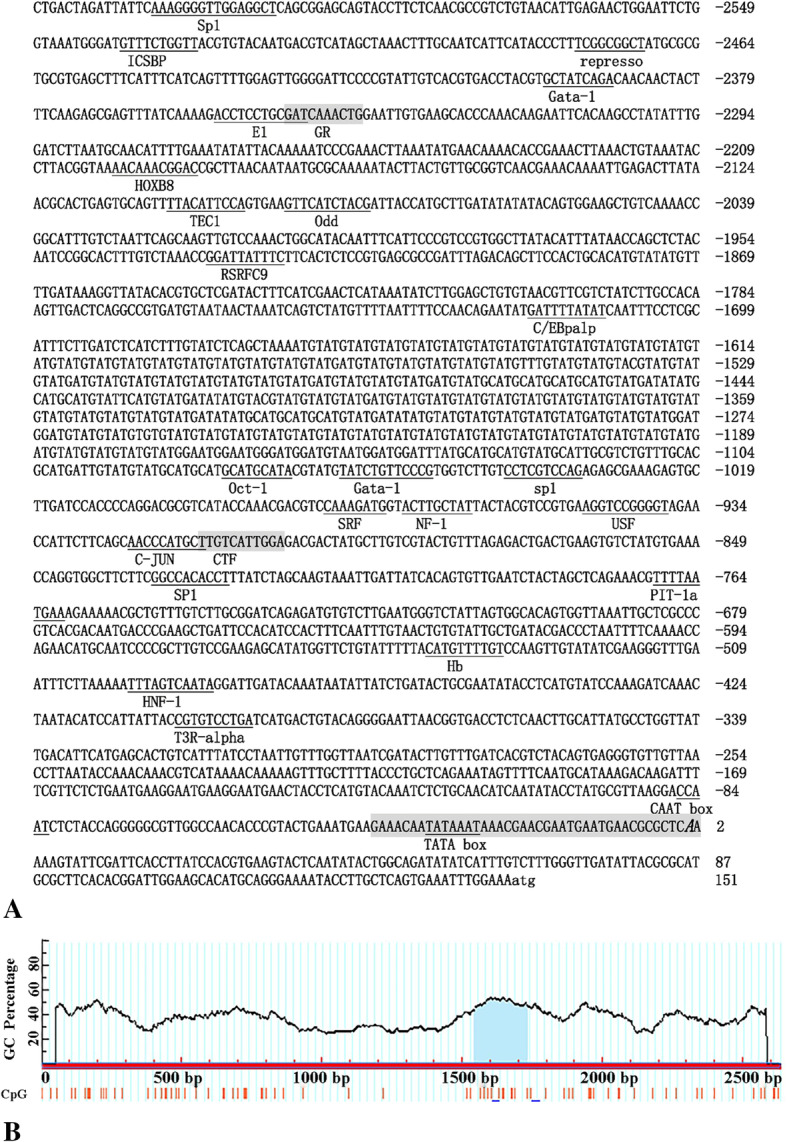
The nucleotide sequence of the 5′-flanking region of *HdHSF1*. **a**. The potential binding sites of the transcription factors are marked with a short, thin line. Overlapping binding sites are indicated by shading. The predicted core promoter region is shaded, the transcription start site in a bold and italic letter, and is located at 1, and the translation start site (atg) is bolded and lowercase. **b**. CpG islands (blue shadow) in the 5′-flanking sequence of *HdHSF1* gene

The 5 ‘flanking sequence (2633 bp) of the *HdHSF1* gene was obtained by Tail-PCR and Genome Walker methods. The bioinformatics analysis showed that the predicted transcriptional start site (TSS) is located at 149 bp upstream of the start codon (ATG), and the core promoter region is located at − 40 bp to + 5 bp when the TSS was specified as 1. The predicted promoter region has a TATA box between − 26 and − 33 bp, a CAAT box between − 82 and − 86 bp, a CpG island with a length of 189 bp (− 902 to − 1090), and many transcription factor binding sites such as GATA-1, NF-1, SRF, Sp1, Oct-1, CTF, C-JUN, and USF are included (Fig. 2).

### Activity analysis of *HdHSF1* promoter in vitro

**Fig. 3:**
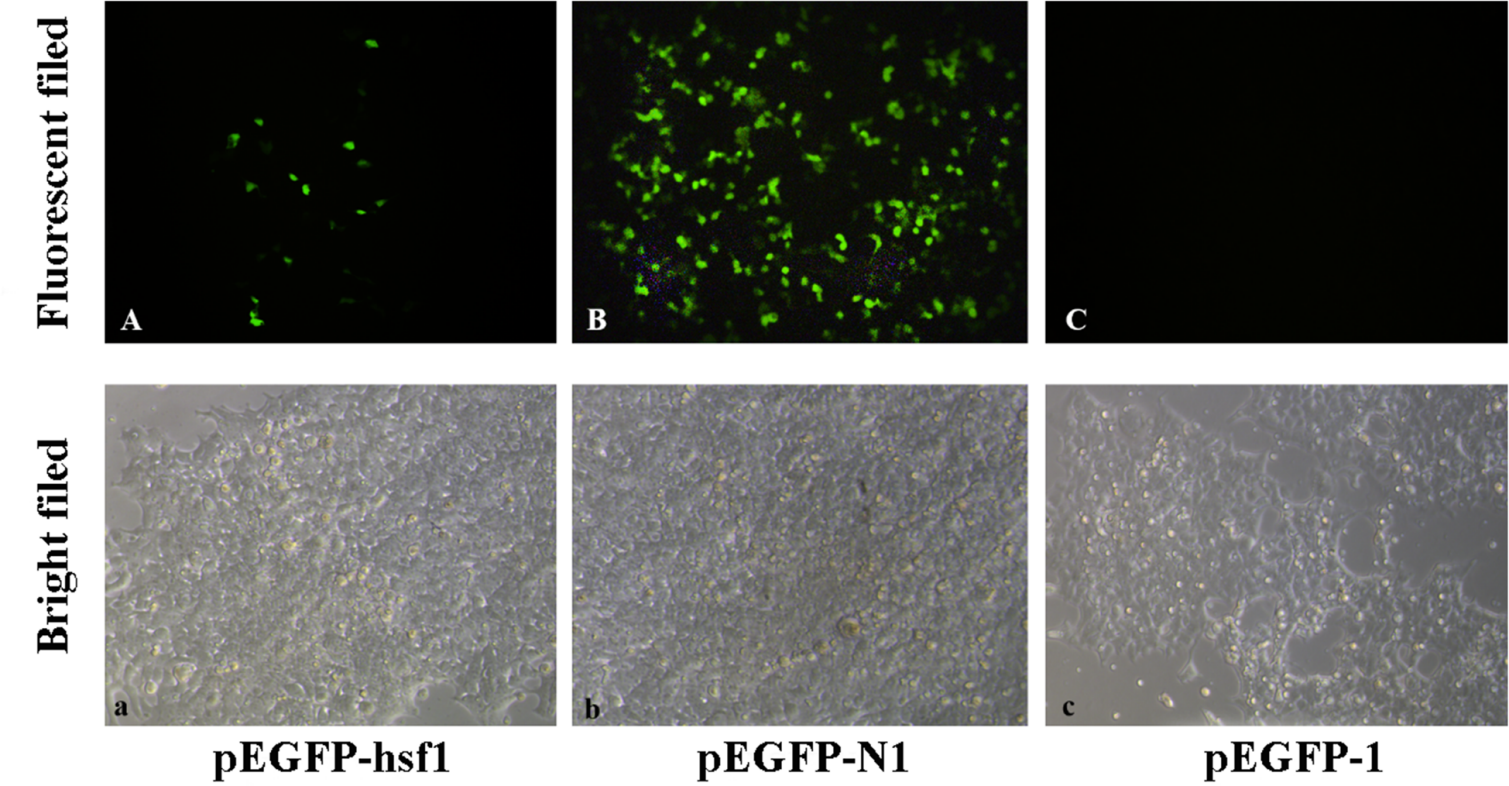
The expression of pEGFP-hsf1 in HEK293FT cells. The EGFP expression of the *HdHSF1* promoter in HEK293T cells at 24 h post transfection with pEGFP-hsf1, which used the *HdHSF1* full-length promoter (**A** and **a**), pEGFP-N1 as a positive control (**B** and **b**) and promoter-less pEGFP-1 as a negative control (**C** and **c**). Fluorescent fields are shown in (**A**, **B**, and **C**), and bright fields are observed in (**a**, **b**, and **c**) separately

To further characterize the promoter functionality of the *HdHSF1* gene, 2633 bp 5′-upstream region was inserted into the pEGFP-1 vector (pEGFP-hsf1) and used to drive the expression of the EGFP gene in HEK293T cells. The pEGFP-N1 promoter used as a positive control had high fluorescence activity as expected. No green fluorescence protein expression was detected in pEGFP-1 as a negative control (Fig. 3).

**Fig. 4:**
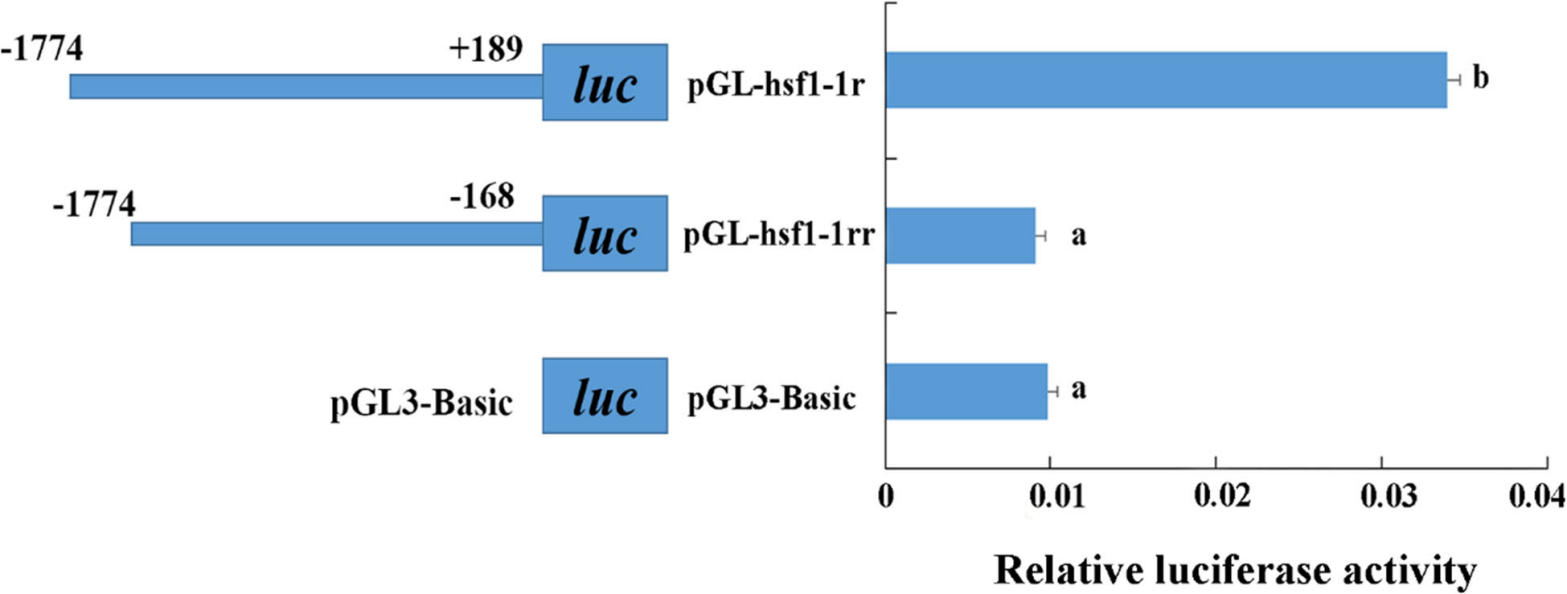
The relative activity of the *HdHSF1* gene with and without the predicted core promoter region. The plasmid containing the core promoter region from − 1774 to + 189 was named as pGL3-hsf1-1r, and the other one lacking the core promoter region from − 1774 to − 168 was named as pGL3-hsf1-1rr. Values are means ± SD of biological replicates (*n* = 3). The significant difference is indicated by a (*) at *p* < 0.05 as compared with the negative control (pGL3-basic). Luc: luciferase expression plasmids

To identify the core promoter region of the *HdHSF1* gene, two constructed reporter plasmids (one containing 1963 bp 5′-upstream region was named pGL3-hsf1-1r; the other one fragment removing the core promoter region was named pGL3-hsf1-1rr) were prepared and transfected into HEK293T cells. The activity of pGL3-hsf1-1r was significantly higher than that of pGL3-hsf1-1rr and negative control (pGL3-Basic, plasmid without insert any target fragments) (*p* < 0.05) (Fig. 4).

**Fig. 5:**
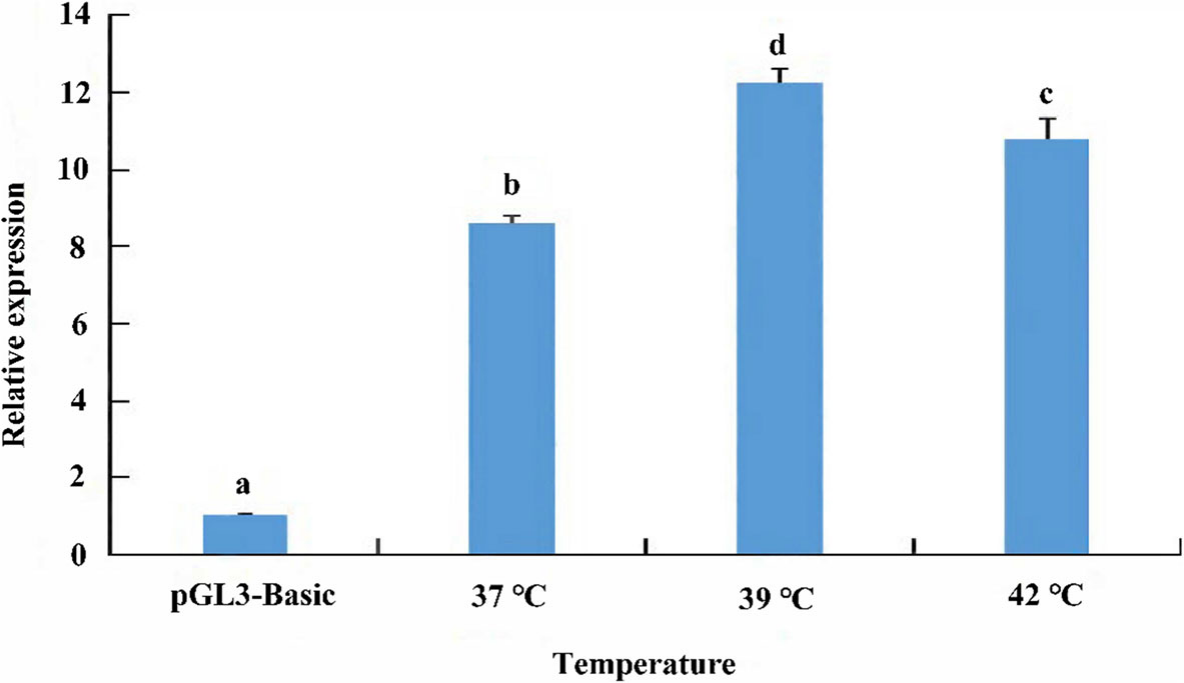
Changes of *HdHSF1* promoter activity in HEK293T cells under high temperature. The cells were incubated at different temperatures (37 °C, 39 °C, and 42 °C) for 40 mins. The means ± SD of biological replicates (*n* = 3) were used to present the relative expression. The pGL3-basic plasmid served as a negative control. The different letters on the error bars represent significant differences, *p* < 0.05

To determine if the expression of the *HdHSF1* gene promoter was induced by heat shock, after we transfected the pGL3-hsf-1r plasmid into HEK293T cells, the cells were incubated at 37 °C, 39 °C, and 42 °C for 40 min. The results showed that under the induction of 39 °C and 42 °C, the luciferase activity was significantly increased (*p* < 0.05), and the activity in 39 °C was the highest (Fig. 5).

**Fig. 6:**
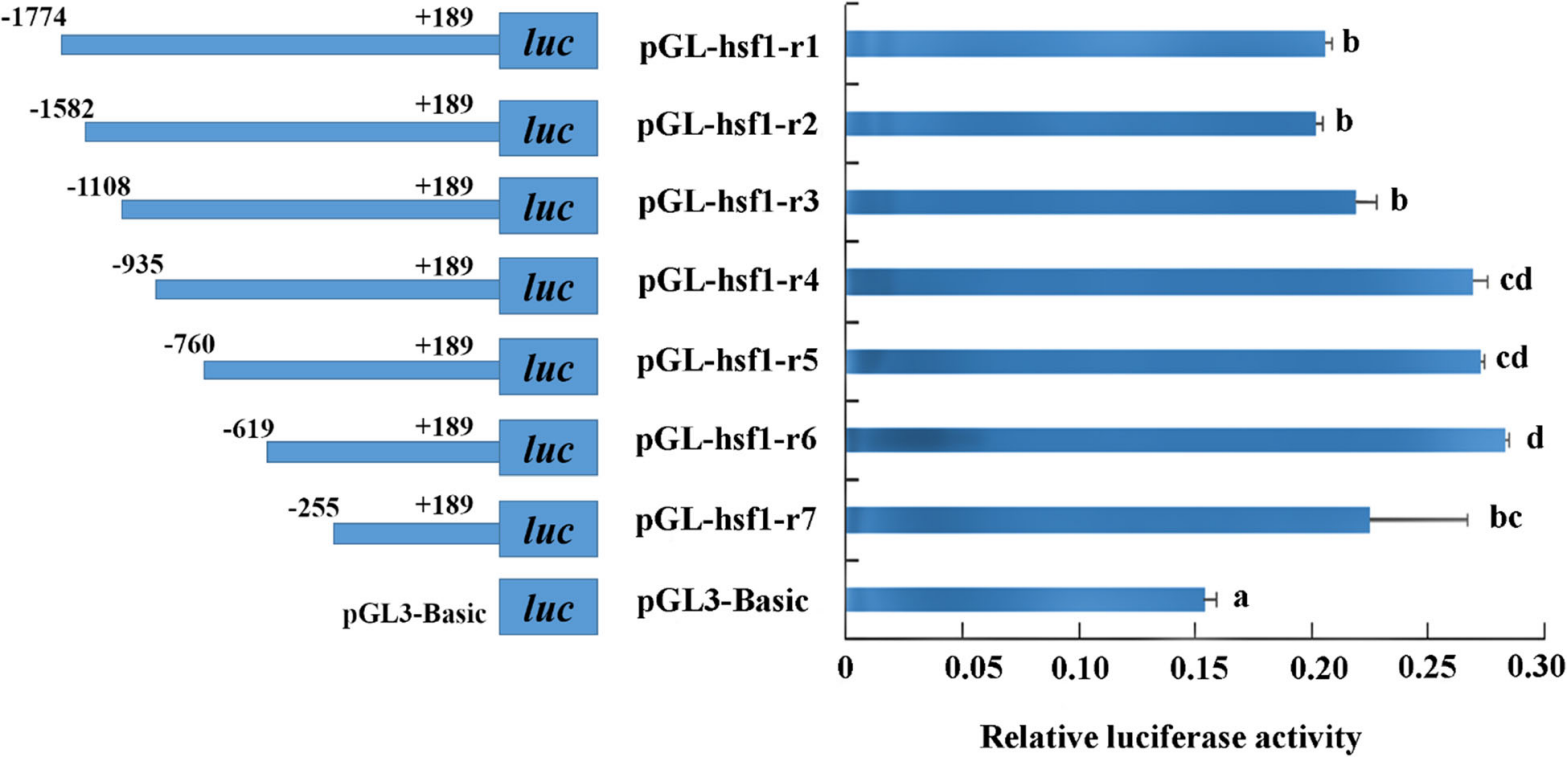
Activity analysis of *HdHSF1* gene promoter in HEK293T cells. Based on the length of the seven fragments containing promoter region, the recombinant plasmids were named pGL3-hsf1-r1, pGL3-hsf1-r2, pGL3-hsf1-r3, pGL3-hsf1-r4, pGL3-hsf1-r5, pGL3-hsf1-r6, and pGL3-hsf1-r7. The pRL-TK vector containing the Renilla luciferase gene was transfected as an internal reference to correct the transfection efficiency. The pGL3-Basic plasmid served as a negative control. The different letters on the error bars represent significant differences, *p* < 0.05. The values are means ± SD of biological replicates (n = 3)

To identify important transcription factor binding sites in the *HdHSF1* promoter region, we transferred a series of different spans of the predicted promoter region containing the transcriptional factor binding element into pGL3-Basic luciferase report vector respectively (named as pGL3-hsf1-r1, pGL3-hsf1-r2, pGL3-hsf1-r3, pGL3-hsf1-r4, pGL3-hsf1-r5, pGL3-hsf1-r6, and pGL3-hsf1-r7). The constructs were used to transfect into HEK293T cells. The results showed that all truncated promoters had detectable activities compared with control (pGL3-Basic, plasmid without insert any target fragments). There were significant differences between pGL3-hsf1-r3 and pGL3-hsf1-r4 or between pGL3-hsf1-r6 and pGL3-hsf1-r7 (*p* < 0.05) (Fig. 6).

**Fig. 7:**
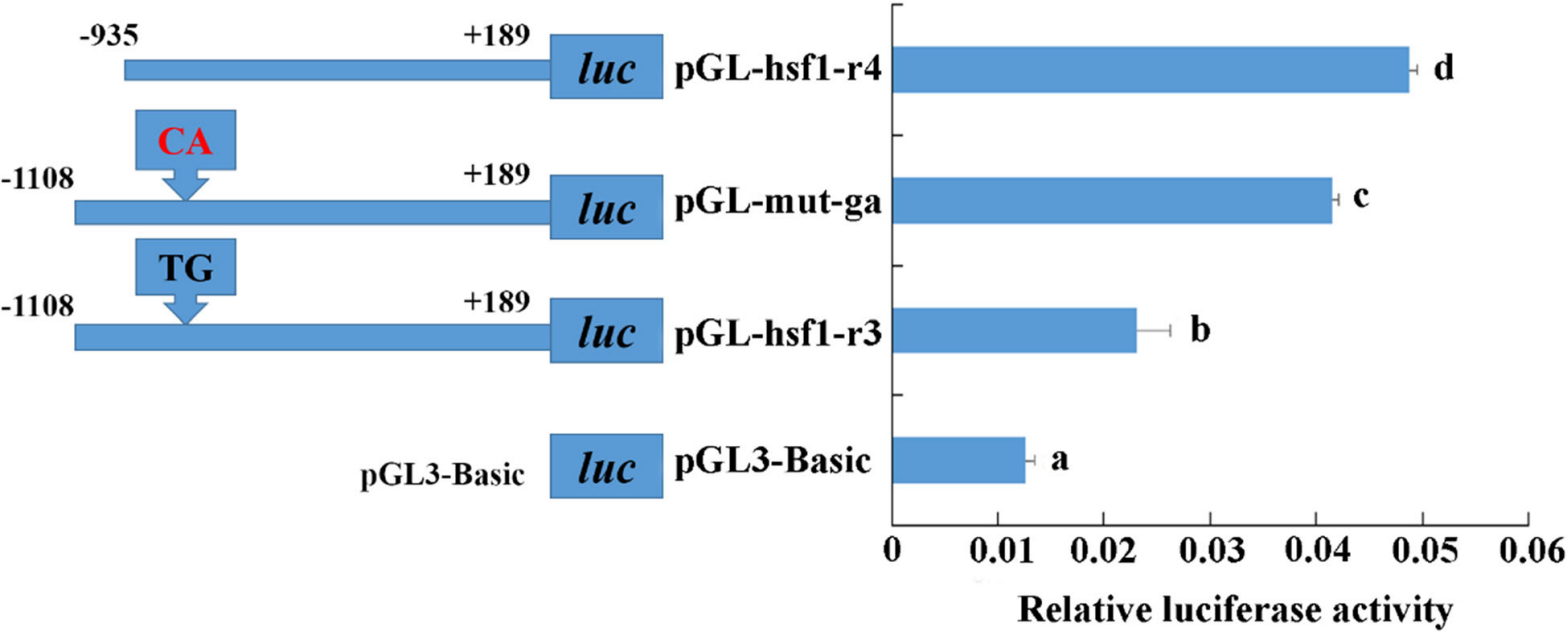
Luciferase activity of the site-directed mutation plasmid pGL3-mut-ga. The distance between pGL3-hsf1-r3 and pGL3-hsf1-r4 contains a sole GATA-1 binding site (ATCTGTTCCC) in the promoter of *HdHSF1*. When TG was mutated into CA, the sequence became ATCCATTCCC, and the mutant recombinant plasmid was named pGL3-mut-ga. The pGL3-basic plasmid was served as a negative control. (The different letters on the error bars represent significant differences, *p* < 0.05). Luc: luciferase expression plasmids

The difference between pGL3-hsf1-r3 and pGL3-hsf1-r4 is the part of − 1108 to -935 bp. There are many predicted transcription factors binding sites in this region, such as Oct-1, GATA-1, Sp1, SRF, NF-1, USF, etc. The TG of the binding site of the transcription factor GATA-1 (ATCTGTTCCC) was mutated into CA (ATCCATTCCC), and the mutant recombinant plasmid was named as pGL3-mut-ga. The results showed that after the gata-1 binding site was mutated, the luciferase activity significantly increased (*p* < 0.05) (Fig. 7).

## Discussion

Diverse stresses, e.g. exposure to heat shock, heavy metal ions, hypoxia, and bacterial infection, have been known to cause denaturation and aggregation of proteins, to disrupt the integrity of essential organelles, and to inhibit vital processes, such as transcription and mRNA translation [1, 22, 23]. The cell response to proteotoxic stresses is mediated primarily through the activation of HSF1 [24]. HSF activates transcription in response to cellular stress. Human HSF1 was proved to contain a central regulatory domain that can repress the activity of its activation domains [25]. A highly conserved DNA-binding domain that can be regulated by interactions between the transcriptional activation domain and the amino-terminal negative regulator might have similar functions in vertebrates and invertebrates [26–29].

Furthermore, the constitutive serine phosphorylation sites were also be proved to have a central role in the negative regulation of HSF1 transcriptional activity by transfected mammalian cells [30]. In the previous studies, the full-length cDNA sequences of *HdHSF1* were cloned successfully [15]. The result of the sequence analysis showed that *HdHSF1* also contained a heat shock factor domain [15], which may be consistent with the functional descriptions in humans [25]. Serine phosphorylation sites that have been reported to have an essential role in the negative regulation of HSF1 transcriptional activity in mammalian cells [30] were also found in *HdHSF1* [15].

The expression level of *HdHSF1* was also demonstrated that it would significantly be up-regulated in gills and hemocytes after heat shock or hypoxia stress to protect cells from damage [15]. It indicated that *HdHSF1* might be involved in the regulation of heat shock response in abalone [15]. Otherwise, HSEs which could be bound by HSFs to mediate HSR and the induction of HSPs were found in the 5′-flanking sequence of *HdHSC70* [20], *HdHSP70* [19], and *HdHSP90* [21] and it indicated that they may all be regulated by *HdHSF1*. So far, the function and regulation of *HSF1* in Mollusca are very limited. This study provides a theoretical basis to *HSF1* regulation mechanisms by cloning, bioinformatics analysis, the transcriptional activity of the 5′-flanking region of *HdHSF1*, and identifying the critical elements involved in its regulation.

### The expression of HSR genes after the inhibition of *HdHSF1*

RNA interference has been proved to be an effective method to study the interaction of different genes. Nowadays, with the rapid development of molecular biological techniques, dsRNA interference has been successfully carried out in *Biomphalaria glabrata* [31], and *H. diversicolor* in our previous study [32–34]. RNAi is initiated by the enzyme Dicer, which cleaves long dsRNA molecules into short double-stranded siRNAs. The well-studied outcome is post-transcriptional gene silencing. The activated RISC-siRNA complex scans, binds, and degrades the complementary target mRNA and leads to gene silencing [35].

To understand the regulation of *HdHSF1* on other genes associated with heat shock in *H. diversicolor*, the *HdHSF1* was transcriptionally inhibited by dsRNA in hemocytes in this study. The qRT-PCR result showed that the expression of *HdHSF1* in the experimental group was significantly lower than the GFP RNAi group and the blank control group, indicating *HdHSF1* was knocked down successfully. After the inhibition of *HdHSF1*, the expression of *HSP22*, *HSP26*, *HSP60*, *HSP70*, *HSP90*, *HSP105,* and *HSBP1* was down-regulated. This result also indicated that *HdHSF1* had a positive regulatory effect on these genes. Although the expression of SIP was significantly up-regulated by thermal stress [18], no significant decrease of *SIP* in response to *HdHSF1* silencing indicated that it might be regulated by other factors and had no relation with the *HdHSF1* gene.

### The *HdHSF1* 5′ upstream sequence

The predicted result by the bioinformatics analysis showed that CpG island, which has been proved to be involved in regulating gene expression, was contained in the 5′-flanking sequence [36, 37]. The predicted result showed that the CpG island of *HdHSF1* was far from the TSS, which was similar to the result of the *HdHSC70* gene but different from that of the *HdHSP70* gene in *H. diversicolor* [19, 20]. The typical CpG islands of eukaryotic genes which can initiate transcription were near or appear on the TSS [38]. Furthermore, the CpG island, which was far from the annotated TSS, has also been indicated to have promoter-like characteristics and was involved in the transcriptional regulation of genes [39].

The TATA box is one of the components in a eukaryotic promoter, which is the most critical binding site of eukaryotic RNA polymerase II, and the sequence pattern is TATAATAAT [40]. Early studies suggested that the TATA box was necessary for the correct transcription of all eukaryotic structural genes. With the development of large-scale genome sequencing, more and more eukaryotic gene sequences were identified, and it was found that there was no TATA box in the 5′ flanks of many genes and elements such as the downstream promoter element (DPE) and the initiator (Inr) could also bound to TFIID in the transcription of core promoters in the absence of a TATA box [41]. In this study, a TATA box was found to be located at 181 bp upstream of initiation codon ATG in the core promoter region of *H. diversicolor* and the loss of this region led to a significant decrease in the activity of the promoter, indicating that *HdHSF1* gene expression was regulated by TATA box.

Regulatory elements are needed for a promoter to sustain transcription in vivo. Transcription factors have to bind to the cis-acting elements to start transcription, no matter they are activators or repressors [42]. Due to the lack of a stable cell line of *H. diversicolor*, the HEK293T cell line, which has been widely used in vertebrate and invertebrate promoter functional analysis [19, 20, 43, 44], was used for the promoter assay in this study. The detection of the promoter activity and the determination of the transcription initiation site were carried out in this study to further characterize the function of the *HdHSF1* promoter. The activity of the complete 2633 bp promoter of *HdHSF1* was verified using the fluorescent expression on the transfected cells with the promoter-EGFP vector (Fig. 3). The luciferase activity decreased significantly in pGL3-hsf1-1rr compared to pGL3-hsf1-1r (Fig. 4) suggested that the core promoter region of the *HdHSF1* gene was located between − 168 − + 189 bp.

HSFs can induce the expression of HSPs by binding to the HSEs present in the promoter regions of HSP genes. So far, the researches on the promoter of the HSFs were limited compared to that of HSPs. Previous results indicated that the HSP genes had an inducible promoter, and the transcription level of these genes significantly increased under high temperatures or other stresses [19, 20, 45]. In this study, the transfected cells were exposed to different temperatures to identify whether the activity of the *HdHSF1* gene promoter was induced by heat shock. The result showed that the luciferase activity of *HdHSF1* had a significant increase after the treatment of HEK293T cells at 39 °C and 42 °C. It indicated that the activity of the *HdHSF1* promoter could be regulated by thermal stress, which is the same as the expression pattern of the *HdHSF1* mRNA in *H. diversicolor* under thermal stress [15]. Nevertheless, although the different high temperatures would cause a change of significant increase in luciferase activity of *HdHSF1*, the activity of *HdHSF1* at 42 °C was lower than that at 39 °C. It indicated that excessive temperature would decrease the activity of the *HdHSF1* promoter, which was similar to the findings in humans [46].

By binding to the binding sites in the upstream region of genes, the positive or negative regulatory transcription factors could regulate the expression of genes [20]. The result of this study showed that all truncated promoters had detectable activities, while a significant difference appeared between pGL3-hsf1-r3 and pGL3-hsf1-r4 (*p* < 0.05). It indicated that a critical transcription factor existed in the deleted site (− 1108 to -935 bp), and it played a central role in the basic transcription of the *HdHSF1* promoter. After mutation in the transcription factor binding site GATA-1 between pGL3-hsf1-r3 and pGL3-hsf1-r4, a certain enhancement effect on the activity of *HdHSF1* promoter was found. Thus, GATA factor may be a negative regulator for *HdHSF1*.

GATA factors are a family of transcription factors that contain a zinc finger. They can recognize the sequence (A/T)GATA(A/G) and are involved in the regulation of gene expression and differentiation [47]. GATA factors have been identified in vertebrates, *D. melanogaster*, *Caenorhabditis elegans*, and plants [47, 48]. The previous study in HL-60 cells demonstrated that the fusion protein p210^BCR-ABL,^ which is a tyrosine kinase that causes transformation and chemotherapy resistance, induces HSP-70 through GATA-1, a trans-factor that binds GATA response element at upstream of HSP-70 promoter [49]. The promoter activity of the fragment with GATA-1 binding sites deletion was significantly decreased. It revealed that GATA-1 could negatively regulate the transcription of the *HdHSF1* gene. However, further research is necessary to clarify the specific regulation mechanism of GATA-1 on *HdHSF1*.

In summary, we demonstrated that HdHSF1 had a positive regulatory effect on other heat shock responsive genes in the small abalone. We cloned and characterized the promoter region of the small abalone *HdHSF1* gene, discovered that GATA-1 was crucial for the transcriptional regulation of the *HdHSF1* gene. It’s the first time to analyze the promoter activity of the *HSF1* gene in Mollusca, and the data might be helpful in further investigate the molecular mechanism of the specific expression pattern of the *HSF1* gene and its regulation on other HSPs to assist in the elimination of irreversibly damaged proteins to resist heat shock or other stresses.

## Conclusions

GATA-1 may be one of the essential transcription factors, which regulate the expression of the *HdHSF1* gene. The inhibition of *HdHSF1* induced the down-regulation of the other *HSP* genes indicated that *HdHSF1* had a positive regulatory effect on these genes. These results suggested that such a possible signal transduction pathway which the transcription factor GATA-1 could regulate the expression of *HSF1* gene and then induced the expression of *HSPs* (except *SIP*) to assisting in the elimination of irreversibly damaged proteins to resist heat shock or other stresses was existed in *H. diversicolor*.

## Methods

### Animals and ethics statement

Adult small abalones (body length 5.88 ± 0.80 cm, weight 16.7 ± 1.80 g) were purchased from the Peiyang abalone farm (Xiamen, Fujian Province). All these abalones were maintained in recycling systems with sand-filtered seawater at a temperature of 25 °C and dissolved oxygen (DO) of 6.2 mg/L as described previously [14–16, 33, 50]. They were fed with sea tangle once a day and held before the experiment. All of the study design and animal experiments were conducted in accordance with the guidelines of Fujian Agriculture and Forestry University’s Animal Care and Use Committee.

### Double-stranded RNA (dsRNA) preparation and exposure assay

To elucidate the regulatory mechanisms of *HdHSF1* on the other heat shock genes, RNA interference was performed by using the dsRNA of *HdHSF1*. The fragment of *HdHSF1* (The full-length cDNA of *HdHSF1* was registered in GenBank with accession No. KC688315) was amplified by PCR using gene-specific primers. The fragment of the GFP gene from the pEGFP-N1 vector was amplified by PCR. The sequences of two pairs of primers were shown in Additional file 1: Table S1. Single-stranded RNA (ssRNA) was transcribed from these PCR products by using T7 phage RNA polymerases (Promega, Shanghai, China). Then DNase I (Promega, Shanghai, China) was used to remove the trace amount of DNA at a ratio of 1 U/μg. After being purified, the sense ssRNA and antisense ssRNA were mixed and annealed at 75 °C for 15 min, at 65 °C for 15 min, and then cool down to the room temperature at the rate of 0.2 °C/s. The formation of dsRNA was monitored by checking the size shift in agarose gel electrophoresis, and the concentration of dsRNA was measured by using a spectrophotometer (NanoDrop ND-1000, Thermo Scientific, Shanghai, China).

The dsRNA of *HdHSF1* was used in the silence experiment. Hemocytes were separately collected by cutting off the foot and were cultured in DMEM medium containing Penicillin-Streptomycin. Then, the hemocytes were divided into three groups:1) Experiment group: *HdHSF1* dsRNA was added directly at a final concentration of 5 μg/ml to the hemocytes culture medium without any vehicle [51]. 2) The control group (GFP RNAi group): GFP dsRNA was added at a final concentration of 5 μg/ml. 3) Blank control group: the medium without any modifications was regarded. There were six replicate beakers of each treatment group, and all samples were incubated at 27 °C for 6 h, and then the hemocytes were harvested to detect the mRNA expression by qRT-PCR.

### Isolation of total RNA and reverse transcription and qRT-PCR verification

Total RNA was extracted by using total RNA Kit II (Omega, Shanghai, China) according to the manufacturer’s protocol. The quality of total RNA was checked by electrophoresis and NanoDrop ND-1000. The cDNA was synthesized in a system including 1 μg total RNA and 2 μL 10 mM random primers by M-MLV reverse transcriptase (Promega, Shanghai, China). The synthesized cDNA was diluted by 100-fold and then stored at − 20 °C until use.

Gene-specific primers for which we want to assay the expression level in RNAi experiment (Additional file 1: Table S1) were used to amplify products of 200–300 bp from cDNA, and the housekeeping *β-actin* gene of *H. diversicolor* (Accession No. AY436644) was selected as the reference gene [14, 16, 33, 52]. qRT-PCR was carried out in a LightCycler480 Roche Real-time Thermal Cycler following the manual with a 10 μL reaction volume containing 4.5 μL of 1:100 diluted original cDNA, 5 μL of 10 × SYBR Green Master Mix (Promega, USA), and 0.25 μL of each primer (10 mM). The cycling conditions for the PCR reaction were set as follows: 1 min at 95 °C, followed by 40 cycles at 95 °C for 15 s, 60 °C for 1 min. Melting curves were also plotted to ensure that a single PCR product was amplified for each pair of primers. The comparative CT method (ΔCT = CT of target gene minus CT of *β-actin* gene and ΔΔCT = ΔCT of any sample minus calibrator sample) for the relative quantification of gene expression was used to calculate the relative expression level of all these genes. Six biological replicates were tested, and each sample was assayed in triplication. The t-test was used to determine the difference in the mean values among the treatments. The difference was considered significant when *p* < 0.05.

### Cloning of the 5′-flanking regions of the *HdHSF1* gene and bioinformatics analysis

The 5′-flanking region of the *HdHSF1* gene was obtained using the Tail-PCR and Genome Walker. The primer sequences used in this study are listed in Additional file 1: Table S1. PCR products were purified and cloned into the pMD19- T simple vector (TaKaRa, Dalian, China), and then sent to Sangon (Shanghai, China) for sequencing.

The putative core promoter region and transcriptional start site (TSS) were predicted using online software, the Neural Network Promoter Prediction (NNPP) (http://www.fruitfly.org/seq tools/promoter.html). The potential important transcription factor binding sites were analyzed by using the AliBaba2.1 (http://www.gene-regulation.com/pub/programs/alibaba2/index.html) database. The CpG islands were predicted by applying the MethPrimer with default parameters (http://www.urogene.org/cgi-bin/methprimer/methprimer.cgi).

### Cell culture, transfection and luciferase assays

The HEK293T cells, obtained from the Eye Institute, Xiamen University, Xiamen, China, were routinely cultured in DMEM high glucose medium supplemented with 8% fetal bovine serum (FBS), 1% penicillin-streptomycin and grew at 37 °C, 5% CO_2_. Transfection experiments were performed in 48-well culture plates. One day before transfection, recipient cells were seeded into wells at a density of 1–3 × 10^5^ cells/well. After removal of culture medium, the cells were transfected with 1 μg of the reporter construct DNA and 0.02 μg of internal reference plasmid in 50 μL Opti-MEM medium per well using 1 μL Lipofectin 2000 (Invitrogen, Shanghai, China) according to the manufacturer’s recommendations. At 24 h post-transfection, the expression of enhanced green fluorescent protein (EGFP) was observed using an inverted fluorescence microscope.

After transfection, the culture medium was discarded, and the cells were washed one to two times in PBS. Each cell sample was then lysed by suspending in 60 μL of 1 × Passive Lysis Buffer (PLB). After centrifugation at 10000 g for 10 min at 4 °C, the supernatant of each sample was taken as 15 μL. The reporter vectors pGL3-Basic (containing a firefly luciferase gene) and pRL-TK (containing a renilla luciferase gene) were obtained from Promega. The activity of firefly luciferase and luciferase of the plasmid were respectively recorded. The luciferase assay was performed using Dual-Glo luciferase assay system (Promega, USA) with pRL-TK vector (expressing Renilla luciferase under herpes simplex virus thymidine kinase promoter) employed as an internal control for normalization of transfection efficiency. The ratio of luciferase activity and the luciferase relative activity was calculated. All the data were obtained from three independent transfection experiments performed in triplicate.

### Construction and transient transfection of the EGFP plasmid

Based on the 5′-flanking region, the most extended 5′-flanking DNA fragment was amplified from the genomic DNA of the *H. diversicolor*. The PCR product was cloned into a pMD19-T vector (TaKaRa, Dalian, China), and then double-digested with KpnI/XhoI enzymes (TaKaRa, Dalian, China) and ligated to pEGFP-1, a promoterless EGFP report vector. The 5′-flanking DNA fragment was located upstream of the EGFP gene. The recombinant vector was named as pEGFP-HSF1. Promoter activity of the 5′-flanking region was then tested by transfecting recombinant plasmid pEGFP-HSF1 into HEK293T cells, the pEGFP-1 and pEGFP-N1 plasmids were served as the negative and positive controls separately. After continuing culture for 24 h, the cells were observed under a fluorescent microscope (Leica Microsystems, Wetzlar, Germany).

### Generation of reporter plasmid constructs

To investigate whether the *HdHSF1* promoter-driven luciferase reporter gene is induced by heat shock, HEK293FT cells were exposed at high temperatures of 37 °C, 39 °C and 42 °C for 40 mins and then their luciferase activities were detected. To produce the luciferase reporter constructs including *HdHSF1* 5′-flanking DNA fragments with different lengths, multiple promoter fragments of the *HdHSF1* gene were generated by PCR and cloned into the pGL3-Basic luciferase reporter vector. Firstly, the universal reverse primers were used in combination with different forward specific primers to create DNA fragments with different lengths and cloned into a pMD19-T simple vector (TaKaRa, Dalian, China). Secondly, the promoter fragment constructs were digested with Kpn I and Xho I, and sub-cloned into Kpn I/Xho I-cut pGL3-Basic reporter vector. Finally, all plasmid constructs were verified by sequencing and purified with an E.Z.N.A.™ Endo-free Plasmid Mini Kit (OMEGA, Shanghai, China) for transfection.

Site-directed mutagenesis (SDM) was a PCR-based approach that can be used to identify the possible function of a specific cis-acting element with primers containing the mutational bases as well as the KpnI and XhoI restriction sites at each of the 5′-terminal separately. It was carried out by overlap extension PCR reactions with similar conditions and procedures, as mentioned above. After determining the transcription factor that may play an essential role in the regulation of gene expression, the interesting fragment with mutagenized cis-acting element was amplified by PCR then purified using a Wizard® SV Gel and PCR Clean-Up System (Promega, USA) and inserted into pGL3-Basic vector containing the recombination sites upstream of the coding sequence of the firefly luciferase. The luciferase plasmid was then constructed. The following program was described as above.

## Supplementary Information

**Additional file 1: Table S1**. Primers used in this article.

## Data Availability

We confirm that the materials described in the manuscript, including all relevant raw data, will be freely available to any scientist wishing to use them for non-commercial purposes, without breaching participant confidentiality.
