# Supplementary material for: Regulatory effect of heat shock transcription factor-1 gene on heat shock proteins and its transcriptional regulation analysis in small abalone Haliotis diversicolor
Source: BMC Mol Cell Biol. 2020 Nov 24;21:83. doi: 10.1186/s12860-020-00323-9 (PMC7685655; doi:10.1186/s12860-020-00323-9)
Supplement: Supplementary file 1 — Additional file 1: Table S1. Primers used in this article. [file 12860_2020_323_MOESM1_ESM.docx]

Additional file 1 Table S1.

Primers used in this article

| Primer name | Nucleotide sequence (5'→3') | Purpose |  |
| --- | --- | --- | --- |
| *HdHSF1-R1* | CAAGACCTCCCGAGCAAA | Genome Walking and Tail-PCR | |
| *HdHSF1-R2* | GAAACTGGTGCCACCCTC |  | |
| *HdHSF1-R3* | CCACCCTGGGTCTTCAACTAA |  | |
| *HdHSF1-R4* | CCACTGTGCCACTAATAGACCC |  | |
| *HdHSF1-R5* | GAAGCCACCTGGTTTCACA |  | |
| *HdHSF1-R6* | AGGACAAGACCACGGGAAC |  | |
| *HdHSF1-F1* | CGGGGTACCACTTTCAAGAGCGAGTTTATC | *HdHSF1* promoter activity | |
| *HdHSF1-F2* | CGGGGTACCGCGGTCAACGAAACAAA |  | |
| *HdHSF1-F3* | CGGGGTACCAGCTCTACAATCCGGCACT |  | |
| *HdHSF1-F4* | CGGGGTACCATGCTTGTCGTACTGTTTAGA |  | |
| *HdHSF1-F5* | CGGGGTACCGAATGGGTCTATTAGTGGCA |  | |
| *HdHSF1-F6* | CGGGGTACCATTATGCCTGGTTATTGACA |  | |
| *HdHSF1-R1* | CCGCTCGAGGCAGGAACAGTTCCAGTATC |  | |
| *HdHSF1-R2* | CCGCTCGAGGACCTCCCGAGCAAACC |  | |
| *HdHSF1-F* | GTGTATGACCAGTCCCGGTT | qRT-PCR | |
| *HdHSF1-R* | CTCCCGGTGACACCTTTCTC |  | |
| *HSP22-F* | GATCGGAGCTAAGCACCTGG |  | |
| *HSP22-R* | TGCTCAACACTCCGTCCTTG |  | |
| *HSP26-F* | GAGCAGACGACAGTCCGAAA |  | |
| *HSP26-R* | TCAGTTTCGCTGTACCCGTC |  | |
| *HSP60-F* | ATGATGAGGAGAGGCTTGCG |  | |
| *HSP60-R* | GTTCGACCGCGGCTATATCT |  | |
| *HSP70-F* | ATCCAGGCCAAGAACGGTTT |  | |
| *HSP70-R* | CCAGCTCCTTCTGCTTGTGT |  | |
| *HSP90-F* | CCTCCCTCCTTGCCTCTGGGT |  | |
| *HSP90-R* | ACACTGTTCATGTACACAATGCGGA |  | |
| *HSP105-F* | TGCCATGGCGGTTGTAGGGA |  | |
| *HSP105-R* | CTGCTGCTTGGCGCCGTATC |  | |
| *HSBP1-F* | GGGAAAGACTACGGTGCAAAC |  | |
| *HSBP1-R* | TATTCTTCTCCAGGTCGTCAA |  | |
| *Sip1-F* | GGAGGACGCCAAGCTTTACA |  | |
| *Sip1-R* | CGGATCCTGCAGGTTAGCTC |  | |
| *β-actin-F* | CCGTGACCTTACAGACTACCT |  | |
| *β-actin-R* | TACCAGCGGATTCCATAC |  | |
| *HSF1-RNAi-F*  *HSF1-RNAi-R*  *GFP-RNAi-F*  *GFP-RNAi-R* | ATCAGTGCCAAGCAGCCGAAGC  AAACGCTGGAGGGGTCACAAGG  GGATCCTAATACGACTCACTATAGGGATC  CTTGTACAGCTCGTCCATGC |  | |
